# Treatment of Epilepsy and Chorea by Partial Tenotomies of the External Muscles of the Eyes

**Published:** 1894-09

**Authors:** 


### *EDITORIAL.*

---

The office of THE JOURNAL is in room 330 Equitable Building.

Address all communications, and make all remittances payable to THE ATLANTA MEDICAL AND SURGICAL JOURNAL, Atlanta, Ga.

Articles for publication should reach this office not later than the 15th instant.

The editors are not responsible for views expressed by contributors.

---

### THE TREATMENT OF EPILEPSY AND CHOREA BY PARTIAL TENOTOMIES OF THE EXTERNAL MUSCLES OF THE EYES.

"If I wished to show a student the difficulties of getting at the truth from medical experience, I would give him the history of epilepsy to read." Such is a quotation from Dr. Oliver Wendell Holmes, which precedes an article by Dr. Casey A. Wood in the New York *Medical Journal* of July 7th, on "The Treatment of Epilepsy by Tenotomy of Eye Muscles and by Other Surgical means." This article by Dr. Wood was very timely, coming as it did when so much is being written by enthusiasts upon the great relief being gained through the operation of partial or total tenotomy of some of the external muscles of the eyes. The real animus of the paper in question was a reply to the article by Dr. A. L. Ranney in the January issue of the New York *Medical Journal,* in which he made reports of numerous cases of epilepsy cured by tenotomy of some one of the external muscles of the eyes. Since the publication of that first article, Dr. Ranney has still further extended his views by publishing in the May issue of the New York *Medical Record* two articles entitled "Eye Treatment of Chorea," in which he advocates the same causes and same treatment as that set forth in his views on epilepsy. Naturally, any seeming new remedy, be it medical or surgical, which is brought forward as a cure for any time-worn disease, as epilepsy, is hailed with delight by all, and hence the articles by Dr. Ranney have attracted a great deal of attention and controversy. If the panacea for epileptics has been found in the remedy claimed by Dr. Ranney, then the mantel of obscurity which has hung around this malady since the days of Hippocrates must be laid aside, and once more must we lay claim to the greatness of the nineteenth century. In the last few years the number of papers written upon the anomalies of the external muscles of the eye, in their relation to certain heretofore unexplained neuroses, is voluminous enough to fill a library. It was to Dr. Geo. T. Stevens, of New York, that we are indebted for bringing the subject prominently before the profession and for instituting a classification and terminology which has simplified the understanding of the subject without indulging in a prolixity of words. I say indebted, because all ophthalmologists must recognize that it is a subject which cannot be disregarded in refractive work, which Dr. Gould says constitutes nine-tenths of ophthalmic practice, and though one may not approach that state of perfectness which is sometimes found by its strongest enthusiasts, yet its importance is being daily brought before the medical profession by men whose standing forbids us to doubt the veracity of their statements. Theories and theoretical men exist in every age, but it must remain for the practical man to give to the public those things which we can call utilitarian. The practical demonstration of any fact is worth more to establish its claim than it can be affected by all the theoretical possibilities hurled against it. If a reputable physician demonstrates by actual clinical cases that such results are obtained by such procedures, then we cannot doubt the truth of the same, although we may be incredulous as to its universal applicability. Because one man has not obtained the same beneficent results as another does not destroy the validity of that obtained by the former. The results obtained by Dr. Ranney, both in the treatment of epilepsy and chorea, are indeed wonderful, but that it is applicable to all cases no one will admit, for the author himself states that he had selected from his case books *some cases* to demonstrate the existence of "eye strain" and extreme forms of "nervous disturbances." The bringing forward of this subject by Dr. Ranney, even though he should be found to be an extremist, will add a new impetus to the subject, thus causing us to gather that which is "chaff" and that which is "wheat." All ophthalmologists have long recognized the relationship existing between "eye strain" so-called and certain nervous phenomena occurring in the same person, the only difference being that some attribute the cause to refractive errors, while others make it dependent upon an unequal balance of the extrinsic muscles of the eyes. To the first category belong the largest number of adherents, because those views are the oldest. It is certainly true, as stated by Dr. Wood in his criticism, that Dr. Ranney did not wait to see the result obtained by correcting the refractive error, but immediately did a tenotomy upon one of the extrinsic muscles, a procedure which was certainly unjust to the refractive error. Space does not permit us to go into a more extended dissertation upon this interesting subject, but we would heartily commend the reading of the articles by Dr. Ranney, of New York, and the criticism by Dr. Wood, of Chicago. If the cases of chorea can be cured by the method as adopted by Dr. Ranney, then we indeed have a boon for many suffering patients. We have seen so many cases of headache and vague neuroses relieved by the proper correction of refractive errors, that one must believe that there is a "modicum of truth" in every extreme measure. We are certainly in full accord with Dr. Ranney when he says "that it is the duty of a physician to have the eyes of all patients afflicted with abnormal nervous disturbances examined early by some oculist who is familiar with and employs the latest methods." R.

